# Are Costs of Robot-Assisted Surgery Warranted for Gynecological Procedures?

**DOI:** 10.1155/2011/973830

**Published:** 2011-09-18

**Authors:** Peter van Dam, Jan Hauspy, Luc Verkinderen, Xuan Bich Trinh, Pieter-Jan van Dam, Luc Van Looy, Luc Dirix

**Affiliations:** ^1^Department of Gynecology, Sint Augustinus Hospital, B2610 Wilrijk, Belgium; ^2^Department of Medical Oncology, Sint Augustinus Hospital, B2610 Wilrijk, Belgium

## Abstract

The exponential use of robotic surgery is not the result of evidence-based benefits but mainly driven by the manufacturers, patients and enthusiastic surgeons. The present review of the literature shows that robot-assisted surgery is consistently more expensive than video-laparoscopy and in many cases open surgery. The average additional variable cost for gynecological procedures was about 1600 USD, rising to more than 3000 USD when the amortized cost of the robot itself was included. Generally most robotic and laparoscopic procedures have less short-term morbidity, blood loss, intensive care unit, and hospital stay than open surgery. Up to now no major consistent differences have been found between robot-assisted and classic video-assisted procedures for these factors. No comparative data are available on long-term morbidity and oncologic outcome after open, robotic, and laparoscopic gynecologic surgery. It seems that currently only for very complex surgical procedures, such as cardiac surgery, the costs of robotics can be competitive to open surgical procedures. In order to stay viable, robotic programs will need to pay for themselves on a per case basis and the costs of robotic surgery will have to be reduced.

## 1. Introduction

The use of robotic assisted surgery has grown exponentially over the last few years as there is a clear trend in surgery, driven by patient demand, to develop less invasive approaches to common procedures [1–7]. Robotic technology has gained popularity in various surgical specialities such as urology, gynecology, thoracic surgery, general surgery, and currently head and neck surgery. The Da Vinci Surgical system is the only FDA-approved robotic system currently on the market [2, 6, 8]. Robot-assisted and laparoscopic surgery eliminates the need for large morbid and less esthetical incisions and often decreases blood loss, postoperative pain, use of pain medication, and length of hospital stay [2, 6]. Advantages of robotic surgery compared to laparoscopy and open surgery are improved dexterity, more precise movements and tremor reduction, and better visualization of the operating field (magnification and 3D). In addition, the robot fingertip hand control mechanism is “intuitive,” meaning that the robotic instruments will move just as your hands move, rather than as a mirror image movement as in laparoscopy [8]. The robotic digital process allows scaling down the surgeons's hand movements to a level at which microvascular or microscopic procedures are feasible. Difficult, minimal invasive surgery is accessible for surgeons without advanced laparoscopic training as it has a short learning curve [6, 9]. Fatigue and frustration become less of a limiting factor for the robotic surgeon compared to the laparoscopic surgeon [10].

The major drawback of the Da Vinci system is the loss of tactile and force feedback. This can be surmounted by training and is partially compensated for by the 3D visual feedback. However, it often leads to rupturing of suture material during knot tying by beginning robotic surgeons. In addition placing the trocars is limited in order to avoid collision of the robotic arms. With the current equipment, this makes it more difficult to operate in the lower and upper abdomen simultaneously [8]. With laparoscopic surgery, the surgeon tends to place the ports in more “natural” and anatomical positions. Esthetically, ports of a laparoscopy are much better located (e.g., the umbilicus and just medial of the anterior superior iliac spine) than the forced trocar placements in an arch, commonly used for robotic procedures. The use of larger trocars (11 mm versus 5 mm) is an additional esthetical disadvantage for robotic surgery compared to laparoscopy. The cart with the robotic arms, which is positioned close to the patient, makes access to the patient limited. Particularly in gynecologic surgery it is sometimes difficult to remove the uterus and other specimens from the vagina after the robot has been docked [2]. Due to the sophisticated technology a robotic team of specialized surgeons, anaesthetists, and dedicated nursing staff is mandatory to make a robotic program function optimally. Particularly, the surgeon and nursing staff need specific training. This makes the use of robotic surgery less practical for nonelective cases. Conventional open surgery, laparoscopic surgery, and robotic surgery require different skills. As the robot is a high-tech complicated instrument to master, adequate training is mandatory before embarking on surgery in patients. It is important to train basic laparoscopic and robotic skills in a box trainer, on a cadaver or on animals [6]. However, the major disadvantage of robotic surgery are the high cost of purchase, maintenance, and instruments of the robotic system. In the current paper we did a systematic review of the literature on the costs of robotic surgery for gynecological procedures.

## 2. Costs of Robotic Surgery

### 2.1. Equipment

There is a major difference in operative costs between open, laparoscopic, and robotic surgery resulting from added expense of specialized equipment. Equipment costs associated with laparoscopic surgery have a relatively low per-case cost as it is multipurpose (e.g., monitors and cameras can be used for laparoscopy but also for hysteroscopy) and can be used by different specialities for many types of surgery [11]. On the contrary, the Da Vinci robot, costing over 1.500.000 Euro and requiring a yearly service contract of 150.000 Euro, has a more limited number of applications. The fixed costs depend greatly upon the number of cases being operated over the amortized life span of the robotic system. We calculated for our unit costs of the hardware (without taking into account disposables) per patient are 3920 Euro, 1960 Euro, 1306 Euro, and 980 Euro if 100, 200, 300, and 400 respectively, robotic procedures a year are performed (amortisation over 7 years). Such costs are not reimbursed by the hospital. The robot also offers a distinct financial disadvantage because each instrument only has a limited preprogrammed (*n* = 10) number of uses, such that the added cost for instruments and drapings can be as high as 1700 Euro per case. A major factor affecting the costs of laparoscopic surgery is the price of laparoscopic instruments. This depends on the type and number of instruments which are used. Generally (semi) reusable instruments are cheaper per case compared to disposable instruments [12]. Although one would expect laparoscopic equipment costs to decrease with time (analogous to the retail computer market), there has been an increase in costs that exceeds inflation despite an increase in the number of procedures performed in most countries [11, 12]. There has been no decrease in the costs of robot-related products due to the lack of market competition.

### 2.2. Operative Times

Operative times play an important role in determining the operative costs. They include the time to start up the procedure, to do the surgery, and to prepare the surgical theatre for the next operation. These costs are calculated in 15–30 min intervals. Costs of anesthesia also increase over similar time intervals. In general, the setup and breakdown of the robotic system take significantly longer compared to the preparation of laparoscopic or open surgery. For many procedures, operative time is lower for open surgery, intermediate for robotic surgery, and slightly longer for comparable laparoscopic surgery [10]. As experience grows with a certain techniques, operative time becomes shorter until they stabilize at a certain level. Lenihan et al. showed that the total operative times for robotic hysterectomies stabilized at approximately 95 minutes after 50 cases [9]. A study that evaluated the learning curve in a series of robot-assisted laparoscopic prostatectomies found that the learning curve may range from a low of 13 cases to a high of 200 cases depending on the surgeon [13]. The average initial time to perform this procedure in this cohort was 424 min, with a final operative time of 230 min per case. The costs of the learning curve are high and can vary widely. For robot-assisted laparoscopic prostatectomies costs of the initial learning cure varied from 49,613 US dollars to 554,694 US dollars with an average of 217,034 US dollars [13, 14]. As in most centers surgeons already went through their learning curves for open and/or laparoscopic surgery the robotic learning curve is an added cost. Operative time in academic institutions, where residents and fellows are trained, may be longer than in private units where the whole surgical team is the same. To overcome these extra costs, the concept of high volume centers is of great importance. In such units the learning curve can be rapidly traversed and costs minimized. Robotic surgery is specifically suitable for virtual reality training, as the operation itself is computer guided. Different companies are developing virtual reality simulators for robotic surgery. This may reduce the learning curve significantly and is likely to be the training of choice for the surgeons of tomorrow [15].

### 2.3. Hospital Stay

Room and board costs represent an important part of the overall cost of hospitalization. For many procedures, the main financial advantage for the laparoscopic and robotic approach is the decreased hospital stay compared to open surgery [11, 16]. The reduced number of inpatient hospital days and earlier return to diet allow for cost savings. These savings can compensate for added expenses in the operating room and result in cost superiority of some procedures. It is important to realize that the costs of hospital beds vary among hospitals, especially between community hospitals and academic medical centers [6]. In addition costs of hospital stay can vary from nation to nation, depending on the health system and the reimbursement by insurances. Although some studies suggest that hospital stay is shorter after robotic surgery compared to laparoscopy [17, 18], for most procedures there was no advantage of a robot-assisted laparoscopic approach over a “pure” laparoscopic approach in terms of rooms and board [19].

### 2.4. Other Costs

In general laparoscopic and robotic procedures allow patients to resume their normal family and professional activities sooner [8, 10]. It is difficult to calculate the savings for society as sick leave, insurance for inability to work, and so forth vary enormously on an individual basis. There is no evidence that long-term morbidity varies considerably among open, laparoscopic, and robot-assisted procedures [16]. 

An interesting advantage of robotic surgery is the more ergonomic position of the surgeon to perform a procedure. Loss of economic productivity of surgeons related to doing laparoscopic and open surgery (e.g., cervical hernia) is a severely underestimated factor. A survey by Matern and Koneczny on this subject shows that 97% of surgeons think improvement of ergonomics in the operating theatre is necessary [20]. In a recent study Tchartchian et al. could demonstrate that robotics improves image stability with fewer corrective maneuvers compared to laparoscopy. Surgeons recorded a significantly higher satisfaction score for the ergonomics of the robot (*P* < 0.001) [21].

### 2.5. Cost Analysis of Robotic Gynecologic Procedures

Sarlos et al. compared the costs of 40 consecutive robot-assisted hysterectomies with 40 matched total laparoscopic hysterectomies. There were no conversions to laparotomy or major morbidity in both groups [22]. Operating time was 83 (55–165) versus 109 (50–170) minutes and hospital stay 3.3 (2–6) versus 3.9 (2–7) days. Average surgical costs were 4067 Euros for the robotic group compared to 2151 Euros in the laparoscopic group. 

Using the Premier Hospital Database Pasic et al. identified women above 18 years of age with a record of minimally invasive hysterectomy performed in 2007 to 2008 [23]. Of 361888 patient records analyzed from 358 hospitals, 95% (*N* = 34527) of laparoscopic hysterectomies were performed without robotic assistance [20]. Inpatient procedures with and without robotics cost 9640 versus 6973 USD, respectively (difference strongly significant). Similar differences were found for outpatient procedures (7920 versus 5949 USD). There were little clinical differences in perioperative and postoperative events. Only surgical times were significantly longer for robot-assisted procedures. 

Barnett et al. used decision modeling to compare costs associated with robotic, laparoscopic, and open hysterectomy [24]. The societal perspective model predicted laparoscopy (10128 USD) as the least expensive approach followed by robotic (11476 USD) and open hysterectomy (12847 USD). In the hospital perspective models laparoscopy was the least expensive (6581 USD) followed by open (7009 USD) and robotic hysterectomy (8770 USD).

Rodgers et al., making a comparison with open surgery, calculated that robotic surgery increased the costs for tubal anastomosis by 1446 US dollars [25]. However Dharia Patel et al. found that the cost per delivery was equal [26]. Robotic rectopexy proved to be 755 USD more expensive than laparoscopic rectopexy [27]. Advincula et al. showed that robotic myomectomy had less complications and shorter hospital stay [28]. They calculated that mean hospital reimbursement was 30.064 USD (SD: 6689) for the robotic procedure versus 13.400 USD (SD: 7720) for open surgery.

Outcomes and costs for endometrial cancer staging via traditional laparotomy (*N* = 40), standard laparoscopy (*N* = 30), and robot-assisted surgery (*N* = 40) were compared in one single institution by Bell et al. [29]. Patients undergoing robot-assisted hysterectomy and staging experienced longer operative times than the laparotomy cohort but with no difference in comparison to the laparoscopic cohort (184 min versus 108 min versus 171 min, *P* > 0.0001, *P* = 0.14). Estimated blood loss was significantly reduced for the robotic cohort in comparison to the laparotomy cohort and comparable to the laparoscopic cohort. The complication rate was the lowest in the robotic group (7.5%) compared to laparotomy (27.5%) and laparoscopic groups (20%) (*P* = 0.015, *P* = 0.03). Average return to normal activity for the robotic patients was significantly shorter than those undergoing laparotomy (24 versus 52 days, *P* < 0.001) and those undergoing laparoscopy (31 days, *P* = 0.005). Lymph node yields were similar in all groups. The total average cost for hysterectomy with staging completed via laparotomy was 12943 USD, for standard laparoscopy 7569 USD, and for robotic assistance 8212 USD.

### 2.6. How Important Are Costs?

Technologic innovation in health care is an important driver of cost growth. Doctors and patients often embrace new modes of treatment before their merits and weaknesses are fully understood [30]. Robotic technology has been readily adopted over the last five years in both Europe and the United States [30–40]. The number of robot-assisted procedures that are performed worldwide has nearly tripled since 2007, from 80000 to 250000 in 2009 [40]. The present review of the literature shows that currently robot-assisted surgery is consistently more expensive than video-laparoscopy and in many cases than open surgery (Table 1). Across the full range of 20 types of surgery for which studies exist the average additional variable cost was about 1600 USD, rising to more than 3000 USD when the amortized cost of the robot itself was included. It seems that currently only for very complex surgical procedures, such as cardiac surgery, its costs can be competitive to similar open surgical procedures [41]. 

It has been suggested that robotic technology may have contributed to the substitution of surgical for nonsurgical treatment for certain diseases [40]. The observed pattern matches evidence from the Surveillance, Epidemiology and End Results Medicare database which shows that Medicare beneficiaries who received a diagnosis of prostate cancer in 2005 were about 14% more likely to have undergone surgery by 2007 than their counterparts whose prostate cancer was diagnosed 3 years earlier [41]. This will probably affect costs on the long run as some studies show that more adjuvant radiotherapy is used after robotic prostatectomy [38]. Barbash and Glied calculated that, if robot-assisted surgery would replace conventional surgeries for the full range of procedures for which cost studies have been done, it would generate nearly 2.5 billion in additional health care costs in the United States [40]. 

The development of new technology and new medications often has a financial motive and the willingness of hospitals and healthcare systems to acquire these advancements often has economic consideration [16]. Hospitals and purchasers of the machines hope of a nice return on investment. Patients only demand the robot because the Da Vinci surgical system is actively marketed to them as “the most effective, least invasive treatment option” [42]. The health care system in many countries is prepared to cover new technologies at higher rates than older technologies, even when there is no proof that the newer technologies provide an additional benefit. Therefore, the crucial question is whether robotic surgery, being more expensive, is better than comparable traditional videoendoscopic and open surgery. Generally, most robotic and laparoscopic procedures have less short-term morbidity, blood loss, intensive care unit, and hospital stay than open surgery [6, 11, 22–39]. Up to now no major differences have been found between robot-assisted and classic video-assisted procedures for these factors in gynecologic procedures [19]. Single exception may be surgical staging of endometrial cancer where it is a consistent finding across all publications that robot-assisted surgery showed less blood loss than laparoscopy. However this difference seems not clinically relevant since it did not have any impact on blood transfusion rate [43].

Most experience on cost calculation for robotics has been gathered in urology. In a recent editorial in European Urology Graefen writes “Are these extra costs justified? Maybe yes, if an advantage for robotic assisted radical prostatectomy over other approaches were documented, but this is currently not the case” [44]. Reviewing the literature on this subject this author concludes that it is clear that high surgical volume is crucial to have a good results, but functional outcome (i.e., continence and erectile function) is not better and in fact significantly more salvage radiotherapy is necessary after robotic surgery [44]. Currently, long-term oncologic outcome is not certain after robot-assisted prostatectomy. Similar echos are coming from the other side of the Atlantic [11]. The exponential use of robotic surgery is not based on evidence-based benefits but mainly patient driven, stimulated by enthusiastic surgeons who love these “high tech toys” and a smart marketing machine created by the manufactures. In order to stay viable, robotic programs will need to pay for themselves on a per-case basis. The advantages of robotics should not be taken for granted and should be further investigated. Multicenter international trials including a health economic section are needed to demonstrate that the higher costs are warranted by superior outcomes. Until then physicians have a responsibility to society and their patients to deliver the best possible care at justifiable cost.

## 3. Future Perspectives

It is crucial for the future of robotics to evaluate the implication of the costs associated with robotic surgery and to analyze what can be done to reduce redundancy and unwarranted costs. Centralisation, specialized robotic surgeons and units, better OR efficiencies, more cases and more competition seem to be the answer. Attempts should be made by the surgeon to minimize the costs of robotic surgery by reducing the number of instruments used (4 instead of 5 instruments: use of only one needle driver saves 292 Euro), reducing the operating time of a procedure (by getting more experience), training dedicated robotic surgeons (not everybody can and should do this surgery in a unit), and stimulating early discharge of the patient when possible (savings will be greater in high cost university hospital versus low cost hospital). The hospital can decrease the costs by increasing the case load by stimulating multidisciplinary use of the robot and centralization of robotic surgery. The annual number of robotic operations should be as high as possible, probably at least 300 procedures. Robotic surgery should preferentially be used for complex surgery. Last but not least, it is of paramount importance that the price of the robot, the maintenance costs, and the price of the drapings and instruments are reduced by the manufacturer in order to keep robotics affordable in most health care systems. Intuitive surgical virtually has a monopoly in robotic surgery and competition is needed in this field. Manufacturers of laparoscopic devices should be creative to make laparoscopic surgery more accessible and to facilitate the use of video-endoscopic surgery, which up to now seems to have a similar efficacy as robotic surgery. However, the future of robotics looks bright as robots will become even smaller and easier to handle, surgeons will get better performing robotic surgery, and eventually robots will become cheaper as almost all electronic devices did when they became more mature with many competitors on the market.

## Figures and Tables

**Table 1:** Cost analysis of gynecologic surgical procedures performed by robot-assisted laparoscopy (RAL), classic video-laparoscopy (CVL), and open surgery (OS). Total hospital costs are given unless specified differently.

Author [reference]	Procedure	RAL	CVL	OS
United States				
Pasic et al. [23]	Hysterectomy			
	Inpatient	9640$	6973$	
	Outpatient	7920$	5949$	
Barnett et al. [24]	Hysterectomy			
	Social perspective	11476$	10128$	12487$
	Hospital perspective	8770$	6581$	7009$
Advincula et al. [28]	Myomectomy	30064$		13400$
Rodgers et al. [25]	Tubal anastomosis			
	Hospital costs	+1446$		
Dharia Patel et al. [26]	Tubal anastomosis			
	Cost per delivery	92488$		92205$
Bell et al. [29]	Endometrial cancer staging			
	(hyst+BSO+LN)	8212$	7569$	12943$

Europe				
Sarlos et al. [22]	Hysterectomy	4067€	2151€	
Heemskerk et al. [27]	Rectopexy	4910$	4165$	
van Dam et al. [41]	Endometrial cancer staging			
	(hyst+BSO+LN)	6707€	4480€	4919€

hyst+BSO+LN: hysterectomy with bilateral salpingo-oophorectomy and pelvic lymphadenectomy.
